# High electronic couplings of single mesitylene molecular junctions

**DOI:** 10.3762/bjnano.6.251

**Published:** 2015-12-18

**Authors:** Yuki Komoto, Shintaro Fujii, Tomoaki Nishino, Manabu Kiguchi

**Affiliations:** 1 Department of Chemistry, Graduate School of Science and Engineering, Tokyo Institute of Technology, 2-12-1 W4-10 Ookayama, Meguro-ku, Tokyo 152-8551, Japan

**Keywords:** break junction, charge transport, mesitylene, single molecular junction, scanning tunnelling microscopy (STM)

## Abstract

We report on an experimental analysis of the charge transport properties of single mesitylene (1,3,5-trimethylbenzene) molecular junctions. The electronic conductance and the current–voltage characteristics of mesitylene molecules wired into Au electrodes were measured by a scanning tunnelling microscopy-based break-junction method at room temperature in a liquid environment. We found the molecular junctions exhibited two distinct conductance states with high conductance values of ca. 10^−1^*G*_0_ and of more than 10^−3^*G*_0_ (*G*_0_ = 2*e*^2^/*h*) in the electronic conductance measurements. We further performed a statistical analysis of the current–voltage characteristics of the molecular junctions in the two states. Within a single channel resonant tunnelling model, we obtained electronic couplings in the molecular junctions by fitting the current–voltage characteristics to the single channel model. The origin of the high conductance was attributed to experimentally obtained large electronic couplings of the direct π-bonded molecular junctions (ca. 0.15 eV). Based on analysis of the stretch length of the molecular junctions and the large electronic couplings obtained from the *I*–*V* analysis, we proposed two structural models, in which (i) mesitylene binds to the Au electrode perpendicular to the charge transport direction and (ii) mesitylene has tilted from the perpendicular orientation.

## Introduction

Along with increasing interests in molecular electronics on the single molecular scale [1], much efforts have been devoted to understand charge transport in a single molecular junction, in which a single molecule is wired to two metal electrodes. In recent years single molecular junctions with electronic functionalities such as diodes [2–8] and transistors [9–13] have been demonstrated. Electronic conductances for most of the single molecular junctions were reported to be below 0.01*G*_0_ (*G*_0_ = 2*e*^2^/*h*, *G*_0_^−1^ ≈ 12.9 kΩ). The low electronic conductances prevent practical application of the molecular junctions for the molecular electronics. To bind a single molecule to metal electrodes, anchoring groups such as –SH [14] and –NH_2_ or (R)_3_–N [15–16] have been used. Such anchoring groups form strong chemical bonds with metal electrodes and the anchoring regions act as resistive space, leading to low electronic conductances of the single molecular junctions. Recently several groups [17–20] including ours [17,21–22] developed a direct π-binding technique, where a π-conjugated molecule is directly bound to metal electrodes without anchoring groups. The direct π-binding technique has been applied for various systems such as benzene derivatives [17,19–20], C_60_ [23–24], ethylene [25], and pyrazine [22,26]. The conductance values of these molecular junctions were close to those of metal atomic contacts. The high electronic conductance is expected to be caused by effective metal–molecule couplings in the direct π-binding to the electrodes. However, there are little direct experimental results revealing strong metal–molecule couplings.

The electronic structure of molecular junctions including metal–molecule couplings can be characterized by current-voltage (*I–V*) characteristics of molecular junctions. Transition voltage spectroscopy [27–28] is one of the methods to characterize energy level of conduction orbitals in junctions by analysing *I*–*V* characteristics. Energy level alignment of conduction orbitals with respect to Fermi level of metal electrodes has been examined for the molecular junctions of alkanedithiol, 1,4-benzenedithiol (BDT) and other molecules [29–31]. Recently *I*–*V* curve analysis [32] based on the Breit–Wigner model [33–37] has been becoming a promising technique to characterize the metal–molecule couplings of the single molecular junctions. Within the Breit–Wigner model, electronic conductance of the single molecular junction can be described by two parameters of (i) the electronic coupling between electrodes and molecule and (ii) the relative energy level alignment of the conduction orbital of molecule with respect to the Fermi level of the metal electrodes. By fitting the experimentally obtained *I*–*V* characteristics to the Breit-Wigner model, the metal–molecule coupling Γ, and energy difference between Fermi level and conduction orbital *ε*_0_ are obtained. This *I*–*V* analysis has been applied for molecular junctions with a variety of anchoring groups. For example Γ = 60 meV and ε_0_ = 0.6 eV have been reported for BDT [37], and Γ = 6 meV and ε_0_ = 0.5 eV for thiolated oligo(phenylene ethynylene) [36].

**Figure 1:**
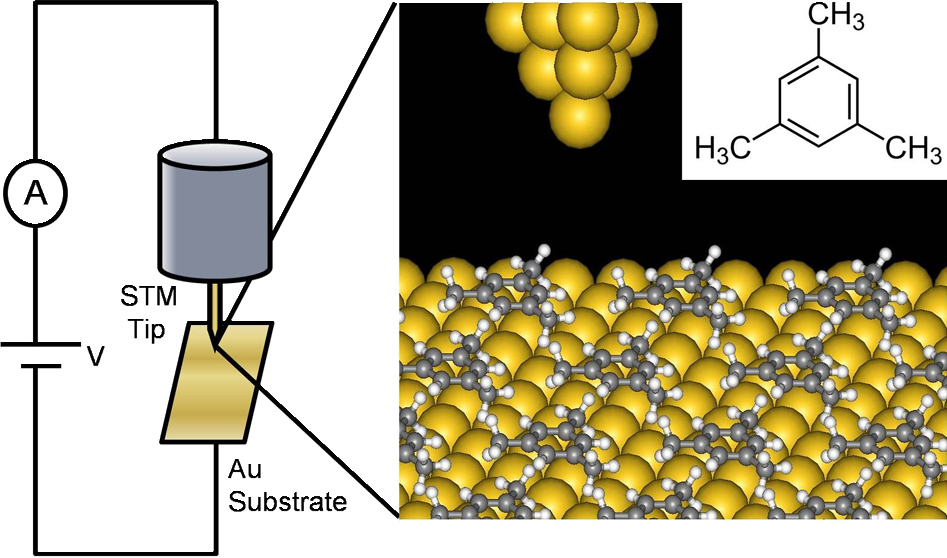
Schematic illustration of the experimental setup. The inset represents structural formula of mesitylene.

In this study, we report on the *I*–*V* analysis for the direct π-bonded molecular junction system without anchoring groups, i.e., mesitylene molecular junctions (Figure 1) to characterize Γ as well as ε_0_. Very recently Afsari et al. have investigated the single mesitylene molecular junction with a scanning tunnelling microscopy (STM)-based break junction (BJ) technique [20]. The conductance of a single mesitylene molecular junction has been founded to be approximately 0.1*G*_0_, which is at least 10 times larger than di-substituted benzene such as BDT [38] and 1,4-benzenediamine (BDA) [16]. The mesitylene molecule can bind to metal electrodes with its molecular plane perpendicular to the direction of charge transport [20]. Here, we investigate the possible origin of the high conductance of the direct π-bonded system. By analysing the *I*–*V* characteristics of the mesitylene junctions, we demonstrate that the direct π-bonded system features larger metal–molecule coupling.

## Results and Discussion

**Figure 2:**
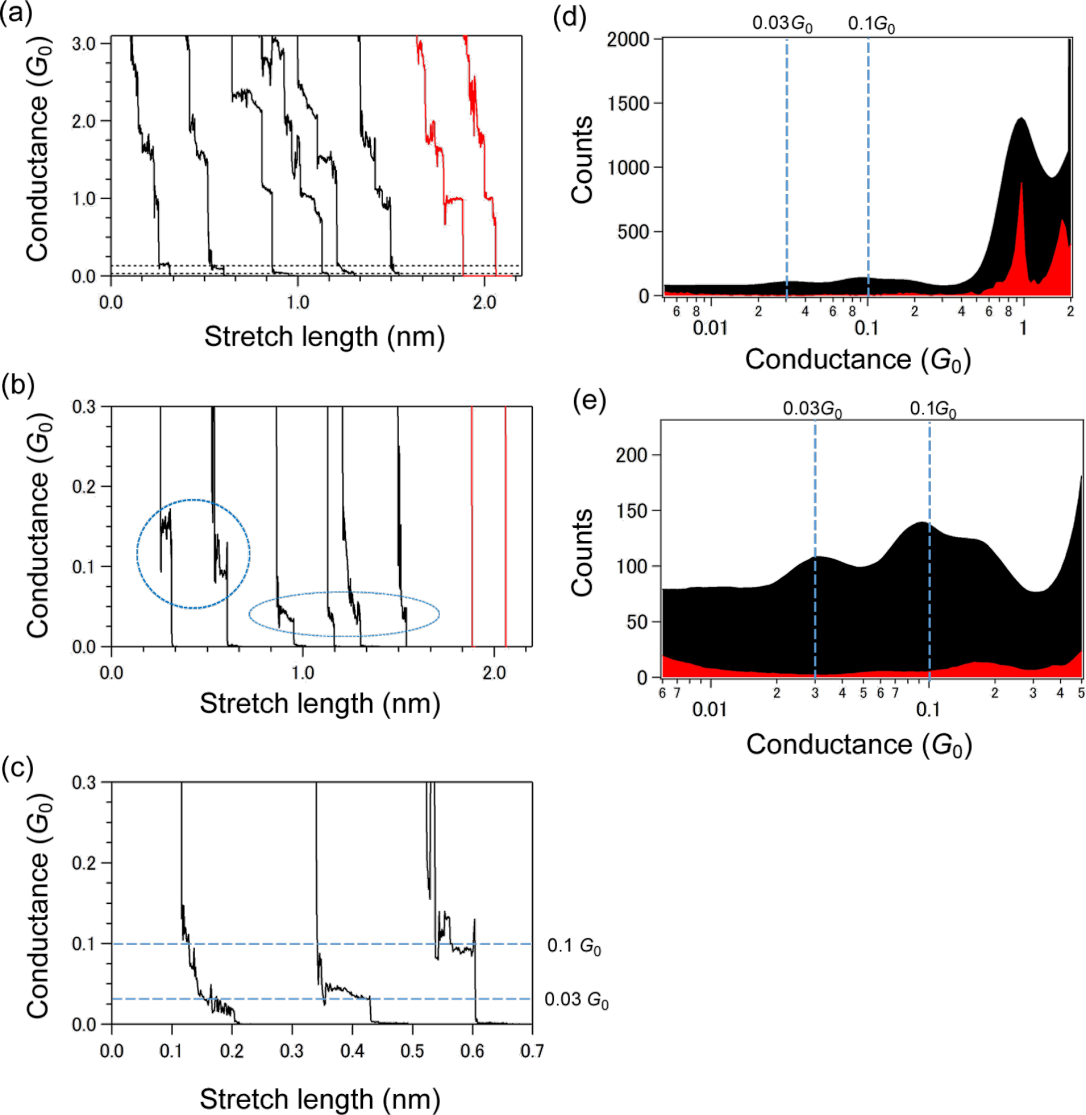
Conductance traces (a–c) and semi-logarithmic conductance histograms (d,e) during STM-BJ rupture process with (black) and without (red) adding mesitylene solution. The bias voltage was set to 20 mV. Conductance windows are 0–3*G*_0_ for (a) and 0–0.3*G*_0_ for (b,c). In (b) traces display high and low conductance steps around 0.1*G*_0_ and 0.03*G*_0_, which are marked by dotted circles. In (c) the two types of steps are magnified. Dotted guide lines are drawn at 0.1*G*_0_ and 0.03*G*_0_ in (c–e). Histograms in (d) and (e) are constructed from a choice of 200 conductance traces. A bin size of Δlog(*G*/*G*_0_) = 0.02 is used.

Mesitylene molecular junctions sandwiched by Au electrodes were prepared by the BJ method [39]. We used the STM-BJ method [15] operating in a liquid environment where thousands of molecular junctions were repeatedly made for statistical analysis of the molecular conductance and *I*–*V* characteristics. Figure 2 shows the conductance traces and histograms during stretching processes of the junctions. In the presence of the molecule, the conductance traces display plateaus at 1*G*_0_, and the corresponding conductance histograms show a peak at 1*G*_0_ (Figure 2a,d). These results indicate that a single Au atomic contact of 1*G*_0_ is formed at the initial stage of the stretching process. This suggests that nano-sized (atomic scale) Au electrodes are repeatedly made just after the rupture of the Au contact to trap a single mesitylene. At the conductance range below 1*G*_0_, conductance steps preferentially appeared around 0.1*G*_0_ and 0.03*G*_0_ (Figure 2b,c), which are absent in blank measurements. Conductance histograms show molecular conductance-peaks around 0.1*G*_0_ and 0.03*G*_0_. We observed two distinct high and low conductance states for a mesitylene junction. In a thousand of measured conductance traces, a small number of traces exhibit switch between the two states. The preferential conductance peaks around 0.1*G*_0_ agrees with the previously reported results by Afsari et al. According to [20], this conductance corresponds to single molecular junctions where mesitylene binds to Au electrodes with its molecular plane perpendicular to the charge transport direction. It should be noted that a side-peak at 0.2*G*_0_ is noticeable in Figure 2e, which is possibly due to formation of multi-molecular junctions. In our conductance measurement, plateaus are also present around 0.03*G*_0_. In STM-BJ experiments, molecular conductance appears as integer multiple plateaus of a fundamental molecular conductance [15] and the fundamental conductance corresponds to the single molecular conductance. Hence, this peak also represents the single molecular conductance of mesitylene junction. In our previous report [40], we have performed STM-BJ experiments on BDA in the mesitylene solution. It has been demonstrated that anime-derivatives form self-assembled adlayer on Au surface [41–42]. In the presence of BDA, both surfaces of the Au tip and Au substrate were covered by BDA in the mesitylene solution. Therefore BDA junctions can preferentially form in the 10 mM BDA mesitylene solution in our previous study. In [20], conductance analysis has been performed using linear-binned histograms and the low conductance state (0.03*G*_0_) found in the present study can be hidden in background tunnelling currents.

**Figure 3:**
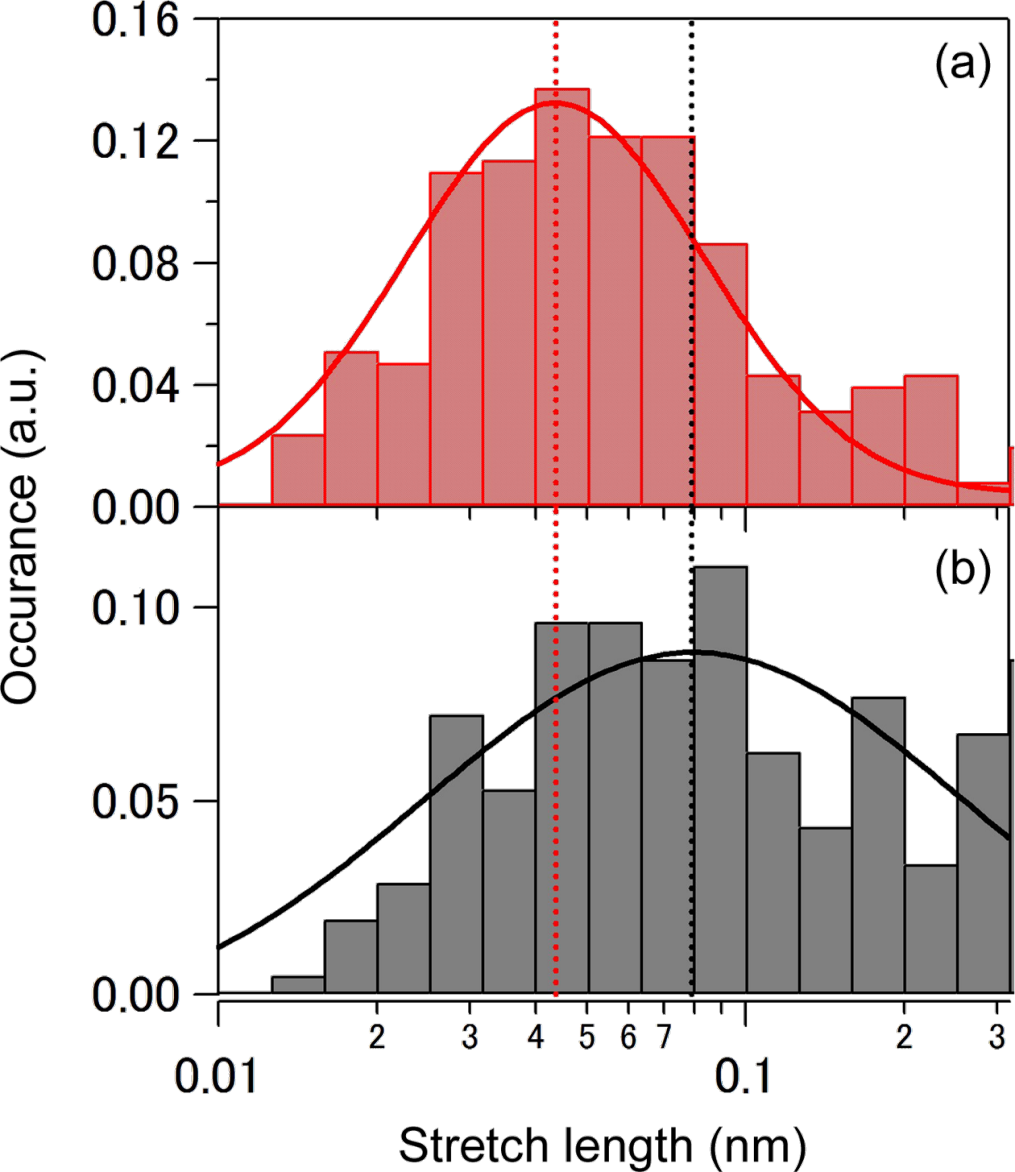
Stretch length histograms on a semi-log scale constructed from 2000 of all conductance traces taken at the bias voltage of 20 mV for (a) high and (b) low conductance states (For a detail see main text). The bin size is log (stretch length/nm) = 0.1. Solid curves are results of Gaussian fits. Peak center positions are indicated by dotted lines ((a) 0.044 nm and (b) 0.079 nm).

To estimate the effective separation of electrodes for the high-conductance and the low-conductance state, we analysed the electrodes separation length in the conductance traces. Figure 3 shows length histograms of the two types of conductance steps. The step length (i.e., the stretch length after the breakage of Au atomic contact) is defined as length of conductance plateau within the conductance range from 0.07*G*_0_ to 0.7*G*_0_ for the high-conductance state and from 0.02*G*_0_ to 0.7*G*_0_ for the low-conductance state. Gaussian fits of the length distribution reveal average step lengths of 0.044 and 0.079 nm for high- and low-conductance states, respectively. The stretch length of the low-conductance state is larger than that of the high-conductance state. It should be noted here that the length does not directly correspond with the actual gap size. The breakage of the Au atomic contact leads to the immediate formation of a nanogap of finite size as a result of the elastic response of the electrode banks. In case of Au atomic contact, the width of the gap thus created is typically 0.4 nm [43]. Considering that mesitylene binds to the Au electrodes with its molecular plane perpendicular to the charge transport direction [20] for the high conductance state of 0.1*G*_0_, the larger stretch length for the low state of 0.03*G*_0_ can be interpreted as a result of tilted orientation of the mesitylene junction upon stretching. We will discuss this matter in a later section.

**Figure 4:**
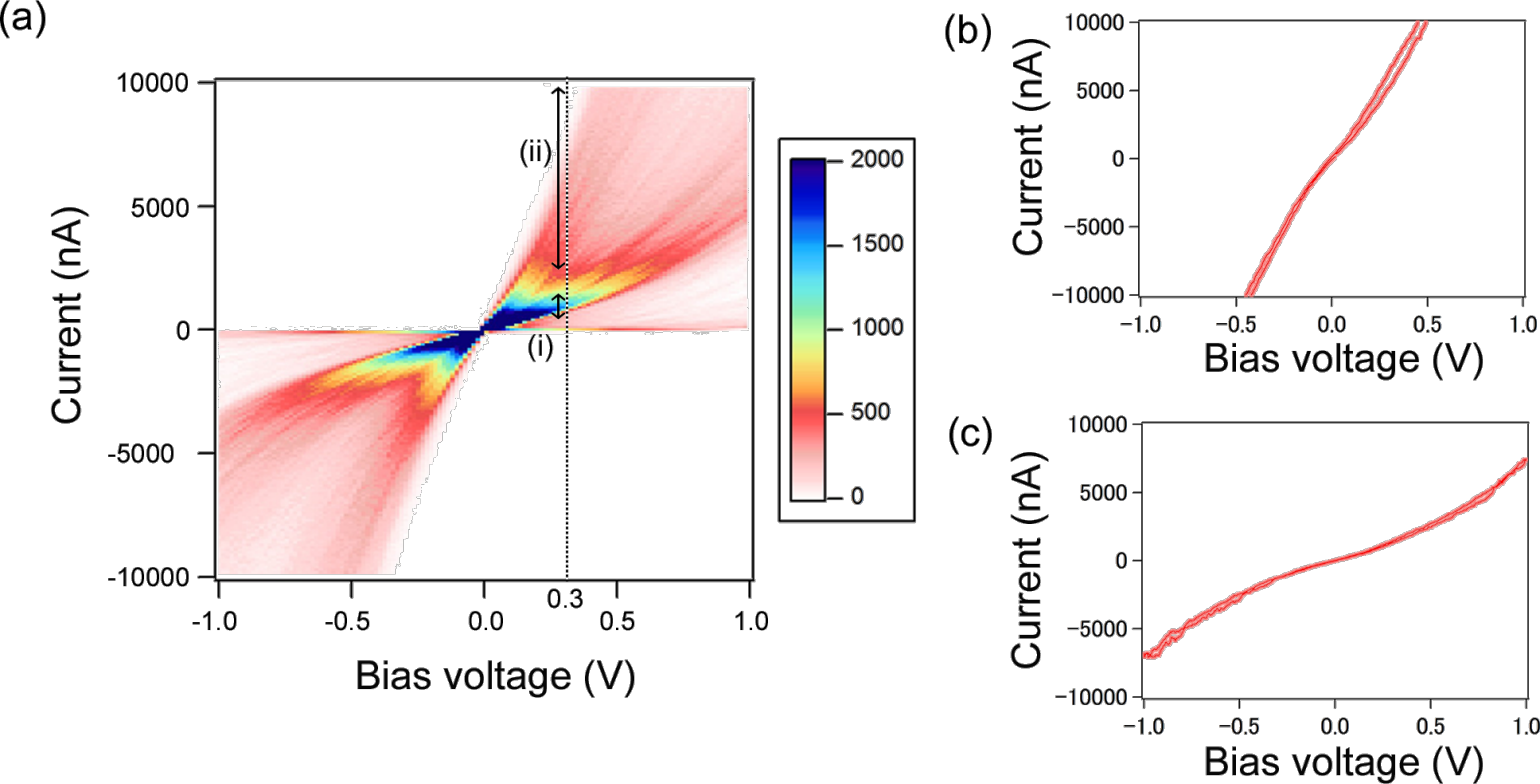
(a) 2D histogram of *I*–*V* curves of the mesitylene molecular junctions. The histogram is built from 1000 *I*–*V* curves. The bin size is 16 mV × 100 nA. Two current windows at 0.3 V are indicated by arrows of (i) and (ii). The current ranges of (i) and (ii) are 700–1500 nA (0.03–0.065*G*_0_) and above 2400 nA (above 0.1*G*_0_), respectively. The conductance windows are chosen to separate *I*–*V* curves into two states with the conductance values of 0.1*G*_0_ and 0.03*G*_0_ observed in Figure 2. The mean current values at 0.3 V are 1100 and 4600 nA in the current windows (i) and (ii), respectively. Deviations are 200 and 1500 nA for (i) and (ii), respectively. (b,c) Typical *I*–*V* curves of the mesitylene molecular junctions in the current windows of (ii) and (i).

We examined *I*–*V* characteristic to obtain the electronic couplings of single molecular junction. In previous research [20], the current-versus-bias relationship of mesitylene junctions has been investigated by repeating current measurement at fixed bias voltages under 0.3 V. It has been reported that the current increases quasi-linearly with the bias voltage at the low bias regime. In this study we measured *I*–*V* characteristics of the mesitylene junctions by sweeping a wide bias range of ±1 V at fixed electrode separations. Figure 4 shows a 2D histogram and typical *I*–*V* curves of mesitylene molecular junctions. In addition to the almost linear increase of the current at low bias voltages, a non-linear increase of the current is apparent at high bias voltages. In the high-bias range, the Fermi level of the Au electrode moves close to the energy levels of the mesitylene conduction orbitals. Thus, the charge transport changes from non-resonant to resonant transport. Two clear distributions are present in the 2D histogram (Figure 4a). This result is in agreement with the two states (high and low sates) in the STM-BJ conductance measurement (Figure 2). To obtain statically averaged *I*–*V* curves for the two states, the current windows of 700–1500 nA (0.03–0.065*G*_0_) and above 2400 nA (above 0.1*G*_0_) at +0.3 V are selected for low and high states, respectively. The conductance windows are chosen to separate the *I*–*V* curves into two states with the conductance values of 0.1*G*_0_ and 0.03*G*_0_ observed in Figure 2.

The transmission of single molecular junction in a single channel resonant tunnelling model is represented by Equation 1 [44].

[1]
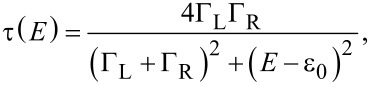


where ε_0_, Γ_L_, and Γ_R_ are the energy alignment of the conduction orbital, and the strength of the coupling between molecule and left and right electrodes, respectively. The current in the molecular junction is represented by

[2]
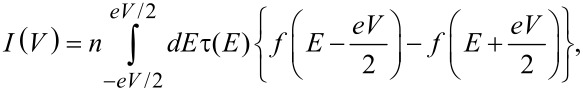


where *n* is the number of bridging molecules. The formula for the current in molecular junctions (Equation 3) is obtained from Equation 1 and Equation 2:

[3]
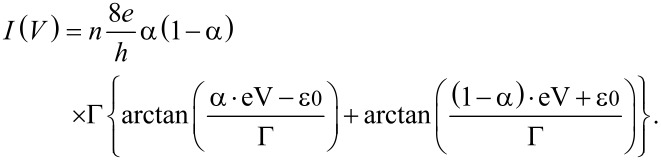


Here, Γ = Γ_L_ + Γ_R_, and α = Γ_L_/Γ. The parameters of electronic structure, Γ and ε_0_, of each states of the mesitylene junction are determined by fitting of the statistically averaged *I*–*V* curves with Equation 3. The fitting results are Γ__H_ = 0.15 eV, ε_0_H_ = 0.31 eV for the high conductance state using bias window ±0.4 V and Γ__L_ = 0.16 eV, ε_0_L_ = 0.72 eV for the low conductance state using bias window ±1.0 V (Γ__L_ = 0.10 eV, ε_0_L_ = 0.49 eV for the bias window ±0.4 V). For both of the high and low conductance states the couplings were found to be symmetric (α = Γ_L_/*Γ* ≈ 0.5). If the high conductance state is due to formation of multi-molecular junctions, we should also observe a fundamental conductance state that comes from the formation of a single molecular junction. Then we checked whether the *I*–*V* curve of the high state can be fitted by multiple formation of the observed low conductance state. The conductance of the high state is 3–4 times higher than that of the low state. The fitting results are Γ__H,n=3_ = 0.098 eV, ε_0_H-n3_ = 0.32 eV for *n* = 3, Γ__H-n4_ = 0.076 eV, ε_0_H-n4_ = 0.37 eV for *n* = 4. The ε_0_H-n3_ and ε_0_H-n4_ are roughly half of ε_0_L_. This mismatch indicates that the high state cannot be explained by multiple formation of molecular junctions. In other words, the high state is not due to the formation of multiple molecular junctions because there is no fundamental conductance state in lower conductance regions for the high state. The *I*–*V* analysis of the statistically averaged data revealed the high and low states have different electronic structures. In contrast to BDT junctions with the anchoring group [37], the coupling of mesitylene junction (Γ = 0.15 eV) is substantially larger than BDT (Γ = 0.06 eV). This result supports the assumption that the molecule binds to the metal via direct π-binding with a strong metal–molecule coupling.

**Figure 5:**
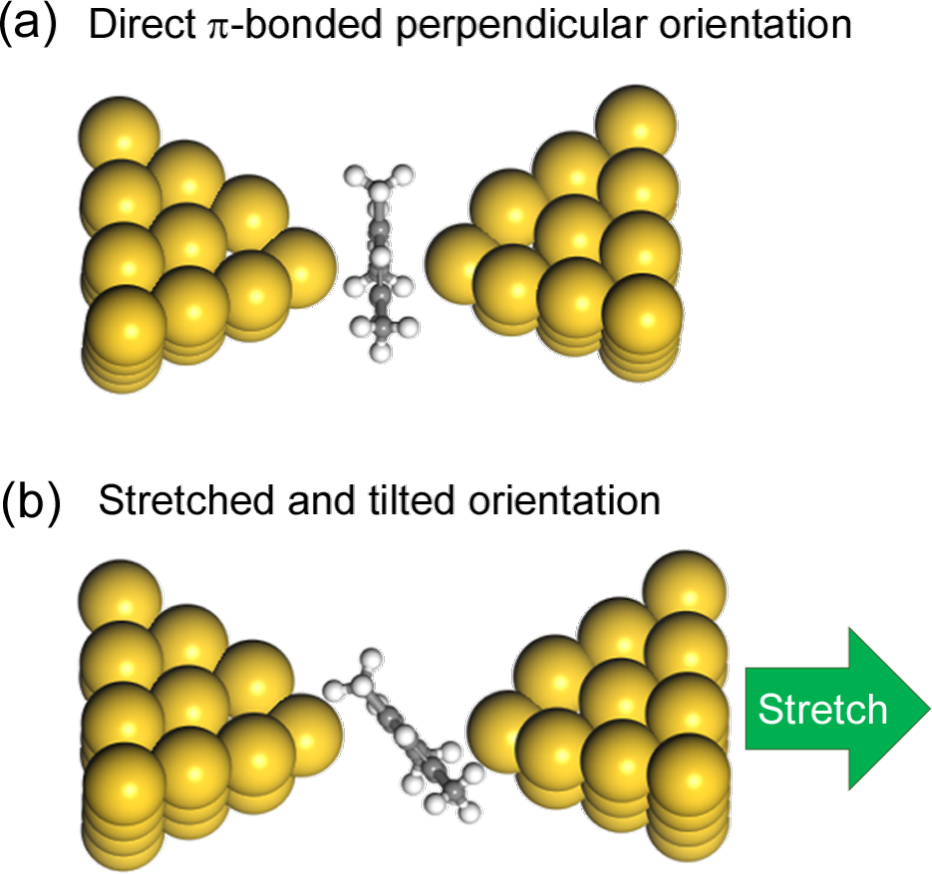
Proposed structural models of the mesitylene molecular junctions for (a) high-conductance and (b) low-conductance states. Mesitylene is oriented perpendicular to the charge transport direction in (a). In (b) stretching of the junction leads to a tilted orientation of the mesitylene molecule. White, grey and yellow balls represent H, C and Au atoms, respectively.

Finally, we comment on structural models for the experimentally observed two states of the mesitylene-molecular junctions. In conductance traces, plateaus corresponding to the low conductance state appear after the plateau corresponding to the high conductance state (Figure 2c). The stretch length analysis of the molecular junctions suggests that the distance between two Au electrodes is larger for the low conductance state than that of the high conductance state. Based on these experimental results, we proposed two structural models for to the high and low conductance states as shown in Figure 5. In the high conductance state, the molecule binds to metal electrode with its molecular plane parallel to the metal electrode (parallel configuration) as proposed previously [20]. Further stretching of the junction in the high state causes tilting of mesitylene and reduction of the conductance in the low state in analogy with benzene molecular junctions [17,19].

## Conclusion

We fabricated single molecular junctions of mesitylene with the STM-BJ method and performed conductance and *I*–*V* measurements. In the conductance measurement and *I*–*V* measurement, two conductance states (a high-conductance and a low-conductance state) were identified. The energy level alignment and the electronic couplings of mesitylene to Au electrodes were determined by fitting of statistically averaged *I*–*V* curves. The origin of the high conductance for the direct π-bonded molecular junctions was ascribed to the experimentally obtained large electronic couplings of ca. 0.15 eV for the two states. Based on the stretch length of the conductance trace and the large electronic coupling obtained from the *I*–*V* analysis, we proposed two structural models.

## Experimental

Conductance measurements were performed using an electrochemical scanning tunnelling microscope (Nanoscope V, Bruker, Santa Barbara, CA). Au(111) substrate was prepared by thermal deposition of Au on cleaved mica surface at 625–675 K. The Au substrate was cleaned by flame annealing before use. Then the Au substrate was fixed in a Teflon liquid cell with the Viton O-ring. Pure mesitylene solvent was poured into the cell. STM-BJ experiments were performed as reported previously. The STM tip was prepared by mechanically cutting Au wire (diameter = 0.3 mm, purity >99%) [45]. In the *I*–*V* experiments, the STM tip was approached to the substrate until a metal junction with conductance of 6.5*G*_0_ was formed in air at room temperature. Subsequently, the tip was withdrawn for 10 nm at a tip speed of 38 nm/s to break the Au junction and to prepare the molecular junction. The tip was held and the bias voltage was simultaneously swept at 100 kHz frequency to record one *I*–*V* curve. The bias voltage range was ±1 V. Then, the junction was broken by pulling away of the tip from the substrate. We repeated this process and obtained more than 1000 *I*–*V* curves. The *I*–*V* characteristics of molecular junction were obtained by removing *I*–*V* curves of Au junctions or vacuum gaps. The *I*–*V* curves for the vacuum tunnelling are defined as *I*–*V* curves with less than 100 nA current at a bias of 1 V (ca. 0.0013*G*_0_) while *I*–*V* curves with less than 10000 nA current at a bias of 0.2 V (ca. 0.65*G*_0_) were assigned to charge transport through metal contacts. More than 95% of the *I*–*V* curves recorded in blank experiments fall into either the vacuum tunnelling or the charge transport through metal contacts.
